# Cook County Hospital

**Published:** 1882-09

**Authors:** 


### COOK COUNTY HOSPITAL.

This institution has just undergone one of those periodical revolutions in its affairs that all institutions under municipal, or more properly, political control, are subject to. For some time differences have been accumulating between the regular medical staff and the County Commissioners, as the governing body. At the meeting of the Board of Commissioners, on the 3d inst., the whole medical and surgical staff was declared vacant, and a new staff was appointed, without the slightest regard for the interests of the three or four medical colleges that have been built up immediately around the hospital, and depending upon it for the important department of clinical instruction. We are assured that the present revolution will not stop with these new appointments, but will soon culminate in putting the hospital under the charge of one medical man as chief, or superintendent, with a salary, and an *advisory board.* This would add another salaried office, with possible *perquisites*, to the number in the county to be scrambled for at every election, and, generally, to be occupied by the doctor who could bring the greatest political influence to the support of the dominant political party, whether he had any medical or surgical reputation or not. Having no personal interest in the present revolution, and no reliable knowledge concerning the causes that led to it, we are not prepared either to defend or censure any one of the parties. But we are prepared to say that a large proportion of the physicians and surgeons so summarily dismissed were as eminent, skillful, and faithful in the discharge of their duties as can be found in the profession anywhere; and that the system of having our public medical charities attended largely by professional men who are willing to *give* their services to the sick for the privilege of giving clinical instruction to those acquiring medical knowledge, is the least liable to abuse or neglect, while the system of *superintendency* under one chief medical officer, with a salary, and directly dependent for his position on political influence and party success, is the most liable to fraud, corruption, and incompetency of any that has ever been tried. And yet we shall not be surprised if the present Board of County Commissioners put it in operation within the next thirty days.

---

### ANEURISMA AORTÆ, COMBINED WITH STENOSIS OF THE TRACHEA AND PARALYSIS OF THE RECURRENT NERVE.

The patient, forty years of age, is a stout man; has had no syphilis, and took sick about seven months ago. There appeared a severe pain under the sternum, the voice became hoarse, and a continued cough troubled him; the breathing was difficult; no pains in the larynx, and no fever. When entering the hospital, the patient was anæmic, and a cyanotic discoloration of the cheeks and mucous membrane was apparent. Dyspnœa in inspiration; temperature subnormal. The peripheral arteries are atheromatous; the larynx projects widely; pulse of the carotids slow; the jugular veins dilated; pressure on the larynx causes no pain. The voice is hoarse; nearly aphonic; the deglutition is not difficult. Thorax long and flat; supra-clavicular grooves deepened; suspiration difficult and hard; respiration twenty per minute. In the anterior part of the thorax is visible a large prominence. A portion between the manubrium, clavicula, right mamillar line and the cartilage of the second rib is somewhat elevated; percussion dull. There is also pulsation coincident with that of the heart. Diaphragm quite low; over the heart dull sounds, also in the fossa pigularis. The patient died from asphyxia. The autopsy showed the aorta enlarged, and there was a sac three cmm. behind the heart, compressing the left bronchus. The recurrent nerve had grown into the aneurism, and the bronchial glands were hardly visible. The trachea was compressed shortly above the bifurcation.—*Med. Chir. Centr. Bl.*

